# Characterization of Fabry mice treated with recombinant adeno-associated virus 2/8-mediated gene transfer

**DOI:** 10.1186/1423-0127-17-26

**Published:** 2010-04-16

**Authors:** Jin-Ok Choi, Mi Hee Lee, Hae-Young Park, Sung-Chul Jung

**Affiliations:** 1 Department of Biochemistry, School of Medicine, Ewha Womans University, Seoul 158-710, Korea

## Abstract

**Background:**

Enzyme replacement therapy (ERT) with α-galactosidase A (α-Gal A) is currently the most effective therapeutic strategy for patients with Fabry disease, a lysosomal storage disease. However, ERT has limitations of a short half-life, requirement for frequent administration, and limited efficacy for patients with renal failure. Therefore, we investigated the efficacy of recombinant adeno-associated virus (rAAV) vector-mediated gene therapy for a Fabry disease mouse model and compared it with that of ERT.

**Methods:**

A pseudotyped rAAV2/8 vector encoding α-Gal A cDNA (rAAV2/8-hAGA) was prepared and injected into 18-week-old male Fabry mice through the tail vein. The α-Gal A expression level and globotriaosylceramide (Gb3) levels in the Fabry mice were examined and compared with Fabry mice with ERT. Immunohistochemical and ultrastructural studies were conducted.

**Results:**

Treatment of Fabry mice with rAAV2/8-hAGA resulted in the clearance of accumulated Gb3 in tissues such as liver, spleen, kidney, heart, and brain with concomitant elevation of α-Gal A enzyme activity. Enzyme activity was elevated for up to 60 weeks. In addition, expression of the α-Gal A protein was identified in the presence of rAAV2/8-hAGA at 6, 12, and 24 weeks after treatment. α-Gal A activity was significantly higher in the mice treated with rAAV2/8-hAGA than in Fabry mice that received ERT. Along with higher α-Gal A activity in the kidney of the Fabry mice treated with gene therapy, immunohistochemical studies showed more α-Gal A expression in the proximal tubules and glomerulus, and less Gb3 deposition in Fabry mice treated with this gene therapy than in mice given ERT. The α-gal A gene transfer significantly reduced the accumulation of Gb3 in the tubules and podocytes of the kidney. Electron microscopic analysis of the kidneys of Fabry mice also showed that gene therapy was more effective than ERT.

**Conclusions:**

The rAAV2/8-hAGA mediated α-Gal A gene therapy provided improved efficiency over ERT in the Fabry disease mouse model. Furthermore, rAAV2/8-hAGA-mediated expression showed a greater effect in the kidney than ERT.

## Background

Fabry disease (OMIM #301500) is an X-linked inborn error of glycosphingolipid metabolism that is caused by a deficiency of α-galactosidase A (α-Gal A) [1]. The lack of this enzyme leads to the progressive accumulation of glycosphingolipids, such as globotriaosylceramide (Gb3) in lysosomes. Gb3 accumulates mainly in the endothelial cells of the kidney, heart, liver, and spleen, as well as in the plasma, and causes diseases such as angiokeratomas, hypohidrosis, stroke, cardiac, and renal failure [2-4].

Enzyme replacement therapy (ERT) with α-Gal A has been developed to treat Fabry disease. Two forms of the enzyme are available: agalsidase alfa and agalsidase beta. Agalsidase alfa (Replagal; Shire Human Genetic Therapies, Cambridge, MA, USA) is produced in a continuous human cell line by gene activation and is used at a dose of 0.2 mg/kg infused intravenously every other week (EOW) [5]. Agalsidase beta (Fabrazyme; Genzyme, Cambridge, MA, USA) is produced in chinese hamster ovary cells and is intravenously administered at a dose of 1.0 mg/kg EOW [6]. These two forms share the same amino acid sequence but have different glycosylation patterns, most likely because of the different manufacturing methods [7]. Clinical trials in adults using both forms of the enzyme have produced biochemical and clinical evidence for their efficacy [6,8,9].

However, potential limitations include the absence of long-term effects using this approach, possible immunological consequences, inevasible progression of renal failure that is impossible to recover, low cost-effectiveness, and overall inconvenience of this treatment as a result of the requirement for continued administration of large doses of enzyme necessary for therapy. Therefore, gene therapy for Fabry disease has been explored using a variety of viral vector delivery systems [10-13]. These gene therapy studies appear to be effective in the Fabry disease mouse model. Among them, a recent gene therapy study using pseudotyped recombinant adeno-associated virus (rAAV) vector showed very promising results [13]. Although the gene therapy studies should have better efficacy and do not have the safety issues compared with clinical use of ERT, there is no report regarding a comparison study of gene therapy and ERT.

In the present study, we investigated pseudotyped rAAV2/8-mediated gene delivery of α-Gal A and compared the efficacy of gene therapy with that of ERT. The AAV serotype 8 capsid was selected because it has shown to transduce mouse hepatocytes better than the AAV serotype 2 [13,14]. Furthermore, comparison of the efficacy of gene therapy and ERT in Fabry mice has focused on their affect on renal pathology, where ERT has been shown to cause the most derangement [9,15].

## Methods

### Animals

A pair of Fabry mice, which were kindly provided by Dr. Roscoe O. Brady of the National Institutes of Health (Bethesda, MD, USA), were bred to acquire a sufficient number of mice for the study [16]. The mice were 18 weeks old at the beginning of the study. All mice were genotyped by polymerase chain reaction (PCR), as described previously [16]. A minimum of three age-matched animals was used for each group. The mice were fed an autoclaved diet and water *ad libitum*. All animals were treated in accordance with the Animal Care Guidelines of the Ewha Womans University School of Medicine (Seoul, Korea). For enzyme replacement therapy, the Fabry mice received an infusion of 1.0 mg/kg body weight of recombinant α-galactosidase A (Genzyme) in normal saline via the tail vein once a week for 6 consecutive weeks [17]. The mice were killed and their tissues were analyzed one week after the last enzyme infusion. The rAAV 2/8-hAGA vector was delivered by intravenous administration via the tail vein of the mice. Blood samples were collected from the tail vein every other week.

### Preparation of rAAV-hAGA viral vectors

The AAV serotype 2-based human α-galactosidase A cDNA containing plasmid harboring the human elongation factor 1-α promoter and the rep2/cap2 or rep2/cap8 plasmids, kindly provided by James M. Wilson, were used to package the expression vector [14]. The rAAV2/2-hAGA and rAAV2/8-hAGA vectors were produced using the triple plasmid transfection method, and purified on a cesium chloride (Sigma-Aldrich, St. Louis, MO, USA) density gradient [12]. The rAAV genomic titer was determined by real-time quantitative PCR using an ABI 7700 TaqMan sequence detection system (PerkinElmer Applied Biosystems, Foster City, CA, USA).

### α-Gal A enzyme activity assay

A fluorimetric assay for α-Gal A was performed as described previously [18] with minor modifications. The tissue samples were homogenized and sonicated in an aqueous buffer containing 5 mg/ml sodium taurocholate, pH 4.4, and centrifuged at 20,000 × *g *for 30 min. The α-Gal A activity was determined by incubating aliquots of the supernatant at 37°C in a pH 4.4 buffer containing 28 mM citric acid, 44 mM disodium phosphate, 5 mM 4-methylumbelliferyl-α-D-galactopyranoside, 4 mg/ml bovine serum albumin and 0.1 M *N*-acetyl-galactosamine, a specific *N*-acetylgalactosaminidase inhibitor.

### Quantitation of Gb3 levels

Extraction and saponification of lipids, and extraction of the glycolytic fraction were performed as described previously [19]. The glycolipid fraction was mixed with 5 ml of *N*-acetyl-galactosylsphingosine and 795 μl of 80% dioxane and then analyzed using a liquid chromatography-mass/mass spectrometer system (LC-MS/MS, ABI 4000; Applied Biosystems, Foster City, CA, USA). Quantitation of glycolipids was performed using a C8 Column and an evaporative light-scattering detector. The Gb3 standard was obtained from Matreya (Pleasant Gap, PA, USA).

### Polymerase chain reaction for the determination of viral vector distribution

Genomic DNA was extracted from livers, kidneys, hearts, spleens, and brains using lysis buffer (100 mM Tris-HCl, 5 mM EDTA, 0.2% SDS, 200 mM NaCl) according to the manufacturer's instructions. Genomic DNA (0.5 μg) using primers and a Power DNA Synthesis Kit (Intron Biotechnology, Seongnam, Korea). PCR amplification was conducted in 20 μl of PCR buffer (50 mM KCl in 10 mM Tris-HCl, pH 9.0 containing 0.1% Triton X; Promega, Madison, WI, USA) containing 0.5 μg of template DNA, 5 μM each of the primers, 0.2 mM dNTP, and 2.5 units of Taq polymerase, for 25 cycles at 94°C for 40 s, at 58°C for 30 s, and at 72°C for 1 min.

### Western blot analysis

Tissue samples (100 mg) that were stored in liquid nitrogen were homogenized in a Pro-Prep solution (Intron Biotechnology, Seongnam, Korea). The tissue lysate was centrifuged at 13,000 × *g *for 30 min, and the supernatant was collected and heated at 100°C for 5 min. Equal amounts of the protein were separated by 8%-12% SDS-PAGE and transferred to a polyvinylidene difluoride membrane (Millipore, Bedford, MA, USA). The membranes were blocked with 5% skim milk in TBST (20 mM Tris-HCl, pH 7.5; 500 mM NaCl; and 0.1% Tween-20) for 2 h at room temperature, and incubated sequentially with the primary antibodies, polyclonal anti-rabbit GLA (α-Gal A) antibody (Santa Cruz Biotechnology, San Diego, CA, USA), or glyceraldehyde-3-phosphate dehydrogenase (GAPDH) antibody (Sigma-Aldrich). The membranes were washed and incubated with the HRP-conjugated secondary anti-rabbit antibodies (Santa Cruz Biotechnology). The washes were repeated two times and the membranes were developed using a chemiluminescent agent (ECL; GE Healthcare, Buckinghamshire, UK) and visualized using a Bio-Imaging analyzer (LAS-3000; Fuji, Tokyo, Japan). The relative protein expression level of the individual genes for each sample was normalized against GAPDH expression.

### Immunohistochemical staining of α-Gal A

The excised tissues were fixed for 24 h in PBS containing 4% paraformaldehyde at 4°C and embedded in paraffin. The sections (4 μm thick) were mounted on silane-coated slides (Muto Pure Chemicals, Tokyo, Japan) and incubated with anti-α-Gal A rabbit antibody (Sigma-Atlas, Stockholm, Sweden) visualized using a Vectastain ABC kit method (Vector Laboratories, Burlingame, CA, USA). The slides were counterstained with hematoxylin and examined using an optical microscope (BH60; Olympus, Tokyo, Japan).

### Immunostaining of Gb3

Mice were anesthetized with ether and perfused through the heart with 0.05 M phosphate buffered saline (PBS), followed by 4% paraformaldehyde (in 0.1 M phosphate buffer). Their kidneys were fixed for 30 min in 4% paraformaldehyde, cryoprotected by infiltration with increasing concentrations of sucrose (10%-30%), and frozen in freezing medium. Kidneys were cut into 5 μm thick sections on a cryostat (CM 3000; Leica Microsystems, Wetzlar, Germany) and collected on gelatin-coated slides. The tissue sections were rinsed in PBS and then immersed in 0.3% hydrogen peroxide (in PBS) for 30 min at room temperature. They were preincubated in 10% normal horse serum (Vector Laboratories) for 1 h and subsequently incubated in rat anti-CD77/Gb3 antiserum (1:200, Chemicon, Temecula, CA, USA) overnight at 4°C. A second incubation with HRP-conjugated anti-rat IgG (1:1000, Vector Laboratories) was performed for 1 h at room temperature. The slides were counterstained with hematoxylin and examined using an optical microscope (BH60; Olympus).

### Ultrastructural study

Mice were killed after 6 weeks of infection with the viral vector. Kidneys were removed and fixed in 10% neutral buffered formalin, methyl Carnoy's solution. For electron microscopy (EM), small blocks of tissues were fixed with 2.5% glutaraldehyde and 2% paraformaldehyde, followed by postfixation in 1% osmium tetroxide, and embedded in Epon using a standard procedure. Epon-embedded blocks were cut at 80 nm with a diamond knife. The ultrathin sections were double-stained with uranyl acetate and lead citrate for electron microscopy. The same block faces were cut at 1 μm with a sapphire knife replacing a diamond knife. These semithin sections were fixed onto lysine-coated slide glasses laying on a hot plate at 60 to 70°C. Ultrathin sections were prepared using a Leica ultratome (Reichert Ultracuts, Wien, Austria) and stained with 4% uranyl acetate for 45 min, and subsequently with lead citrate for 4 min at room temperature. Sections were examined in an H-7650 electron microscope (Hitachi, Ibaraki-ken, Japan).

### Liver function test

Hepatic toxicity marker enzyme activities, alkaline phosphatase (ALP), serum glutamic oxaloacetic transaminase (SGOP), and serum glutamic pyruvic transaminase (SGPT) in the serum were measured using standard protocols [20].

### Statistical analysis

The statistical significance of differences between groups was determined using an ANOVA with Student's *t *test. Null-hypothesis probabilities of *p *< 0.05 were considered significant. All values are expressed as means ± SD.

## Results

### Distribution of recombinant adeno-associated virus vectors in mouse tissue

**Figure 1:**
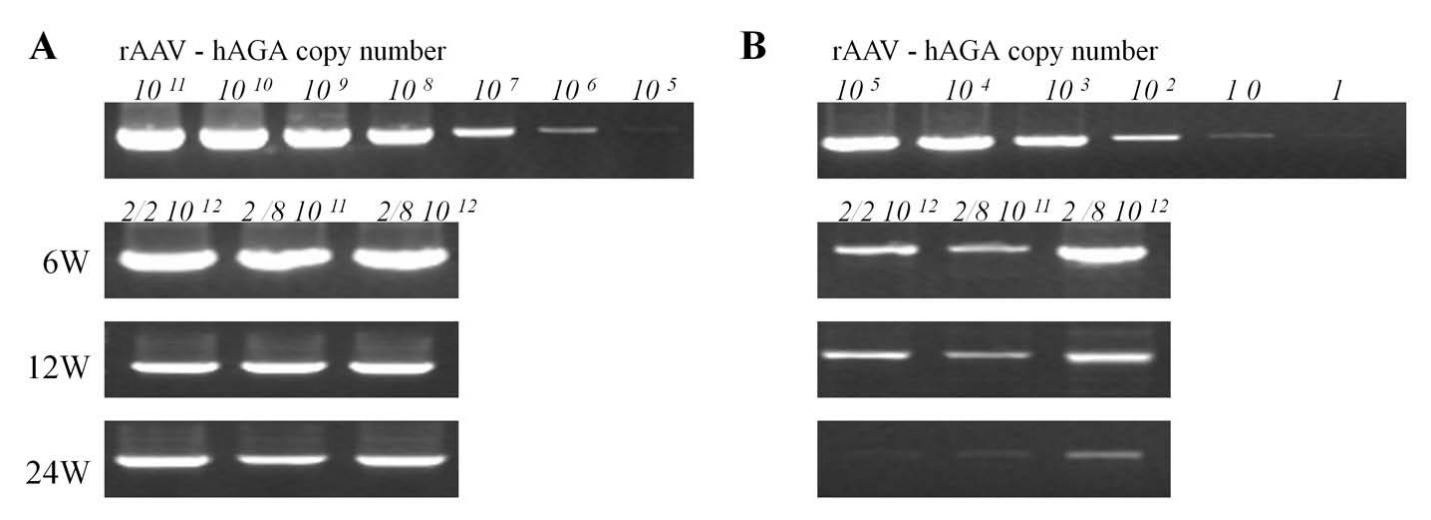
**PCR analysis of transduced α-Gal A gene in Fabry mice**. DNA was extracted from the organs of Fabry mice 6, 12, and 24 weeks after vector injection and analyzed by PCR. rAAV-hAGA, whereas the 1.4-kb fragment corresponds to the mouse genomic α-Gal A gene. The distribution was identified in liver (A) and kidney (B) at 6, 12, and 24 weeks after treatment.

The distribution of the rAAV-hAGA vector was assessed by isolating genomic DNA and determining the viral genome sequence in the liver, kidney, heart, spleen, and brain of Fabry mice injected with 2 × 10^12 ^particles of rAAV 2/2-hAGA, 2 × 10^11 ^particles of rAAV 2/8-hAGA, or 2 × 10^12 ^particles of each rAAV 2/8-hAGA vector. The genomic dosage of the viral vector was identified at 6, 12, and 24 weeks after tail-vein injection. Quantitative analyses revealed a dose-dependent increase in the copy number of rAAV-hAGA in the liver (Fig. 1A) and kidney (Fig. 1B).

### α-Gal A activities in Fabry mice treated with rAAV-AGA vector

The α-Gal A enzyme activity was determined in the liver, kidney, heart, spleen, and brain of mice at 6, 12, and 24 weeks after injection of rAAV-hAGA via the tail vein (Table 1). The average α-Gal A enzyme activity in the liver of wild-type mice was 75.7 ± 29.3 nmol/mg protein. Fabry mice injected with 2 × 10^11 ^particles of rAAV 2/8-hAGA and 2 × 10^12 ^particles of rAAV 2/8-hAGA vectors showed α-Gal A activities of 1,861.4 ± 45.2 nmol/mg protein and 2,137.5 ± 80.9 nmol/mg protein, which were 24 times and 30 times that of wild-type mice at 6 weeks after treatment, respectively. α-Gal A enzyme activities of 423.2 ± 24.5 nmol/mg protein and 1,267.6 ± 30.8 nmol/mg protein were also observed in the kidney and were 7 and 20 times that of the wild-type mice at 6 weeks after treatment. In the heart, spleen, and brain, the α-Gal A activity was significantly higher in treated Fabry mice than in wild-type mice. At 12 and 24 weeks after treatment, the α-Gal A enzyme activities were still significantly higher in the tissues of treated Fabry mice than of wild-type mice. The α-Gal A activities in the liver, kidney, and spleen were maintained for up to 60 weeks postinjection. These results were compared with those of Fabry mice that received ERT. The α-Gal A enzyme activity in the mice treated with 2 × 10^12 ^particles of rAAV 2/8-hAGA was significantly higher than that in the Fabry mice that received ERT. These results demonstrated that the rAAV 2/8-hAGA vector was efficiently expressed in liver and kidney and that it produced high levels of α-Gal A.

**Table 1:** α-Gal A enzyme activity in the tissues of mice after tail vein administration of rAAV2/8-hAGA vector

Mice group	Weeks after injection	Enzyme activity (nmol/h/mg protein)				
		
		Liver	Kidney	Spleen	Heart	Brain
Wild-type mice		75.7 ± 29.3	63.6 ± 20.5	189.7 ± 23.2	55.1 ± 7.18	111.8 ± 12.2

Fabry mice		1.2 ± 0.13	1.3 ± 0.47	4.3 ± 0.97	0.3 ± 0.16	1.4 ± 0.26

Treated mice 2 × 10^11 a^	6	1861.4 ± 45.2**	423.2 ± 24.5***	2036.1 ± 47.0**	1837.2 ± 40.1***	106.9 ± 8.1
	12	186.1 ± 65.7*	161.7 ± 62.1*	1059.9 ± 423.7**	1263.0 ± 152.0**	66.3 ± 3.2
	24	90.8 ± 42.7	19.4 ± 0.5	305.9 ± 14.1	31.4 ± 4.04*	10.0 ± 2.1

Treated mice 2 × 10^12 b^	6	2137.5 ± 80.9***	1267.6 ± 30.8***	6413.8 ± 336.9***	4614.7 ± 179.8***	310.3 ± 7.5***
	12	1062.3 ± 189.8***	600.6 ± 392.2***	4276.1 ± 214.1***	1297.9 ± 746.0***	263.0 ± 87.2*
	24	734.4 ± 79.2***	270.1 ± 4.5**	1216.3 ± 44.8***	601.6 ± 13.2***	257.0 ± 18.6*
	48	366.2 ± 22.8***	127.0 ± 14.7**	451.0 ± 9.7**	81.1 ± 10.3**	247.2 ± 30.3*
	60	245.5 ± 95.0***	64.0 ± 23.7	335.7 ± 11.8**	23.2 ± 1.3	37.7 ± 9.2

Treated mice ERT^c^	6	84.3 ± 15.4*	30.7 ± 7.5	96.7 ± 26.5	67.18 ± 4.6*	142.4 ± 37.6**

Data are presented as average ± SD, ^a^mice treated with 2 × 10^11^: rAAV2/8-hAGA (2 × 10^11 ^particles/mouse). ^b ^mice treated with 2 × 10^12^: rAAV2/8-hAGA (2 × 10^12 ^particles/mouse), ^c ^ERT treated mice: 1.0 mg/kg once a week for 6 consecutive weeks. * *p *< 0.05, ** *p *< 0.01, and *** *p *< 0.001 vs. wild mice (paired *t *test), *n *= 3.

### Gb3 levels in Fabry mice treated with rAAV-hAGA vector

The levels of Gb3 in the liver, kidney, heart, spleen, and brain of treated Fabry mice were determined at 6, 12, and 24 weeks after injection (Table 2). After the injection of 2 × 10^11 ^particles of rAAV 2/8-hAGA vector, there was decrease in the Gb3 level in the liver, kidney, spleen, and heart at 6 weeks, whereas the Gb3 content in the brain was reduced moderately after 24 weeks. The Gb3 levels in the tissues were dramatically decreased after injection of 2 × 10^12 ^particles of rAAV 2/8-hAGA vector. However, Gb3 reaccumulated in the kidney and brain at 24 weeks after the injection.

**Table 2:** Gb3 levels in mouse tissues after tail vein administration of rAAV2/8-hAGA vector

Mice group	Weeks after injection	Gb3 levels (nmol/mg protein)				
		
		Liver	Kidney	Spleen	Heart	Brain
Untreated Fabry mice		2.498 ± 0.261	7.466 ± 0.743	20.665 ± 5.999	7.179 ± 1.939	2.079 ± 1.099

Treated mice 2 × 10^11a^	6	0.001 ± 0.001	0.032 ± 0.018	0.014 ± 0.007	0.003 ± 0.001	0.139 ± 0.022
	12	0.010 ± 0.001	0.832 ± 0.108	1.098 ± 0.129	0.058 ± 0.047	0.520 ± 0.192
	24	0.015 ± 0.001	1.948 ± 1.487	1.392 ± 0.215	0.054 ± 0.040	1.832 ± 0.299

Treated mice 2 × 10^12b^	6	0.005 ± 0.002	0.019 ± 0.007	0.013 ± 0.006	0.002 ± 0.001	0.092 ± 0.043
	12	0.007 ± 0.004	0.640 ± 0.349	0.197 ± 0.054	0.019 ± 0.076	0.325 ± 0.146
	24	0.019 ± 0.020	0.359 ± 0.011	0.695 ± 0.252	0.040 ± 0.019	1.267 ± 0.210
	48	0.046 ± 0.001	0.008 ± 0.001	0.732 ± 0.026	0.069 ± 0.108	1.434 ± 0.097
	60	0.092 ± 0.001	0.023 ± 0.071	0.969 ± 0.049	0.502 ± 0.901	1.640 ± 0.127

Treated mice ERT^c^	6	0.002 ± 0.001	0.263 ± 0.062	0.015 ± 0.005	0.003 ± 0.001	0.098 ± 0.025

Wild-type mice		0.036 ± 0.013	0.150 ± 0.013	0.252 ± 0.058	0.036 ± 0.0087	0.025 ± 0.011

Data present as average ± SD, ^a ^mice treated with 2 × 10^11^: rAAV2/8-hAGA (2 × 10^11 ^particles/mouse), ^b ^mice treated with 2 × 10^12^: rAAV2/8-hAGA (2 × 10^12 ^particles/mouse), *n *= 3. ^c^ERT treated mice: 1.0 mg/kg once a week for 6 consecutive weeks.

### α-Gal A expression in the liver and kidney of the Fabry mice

**Figure 2:**
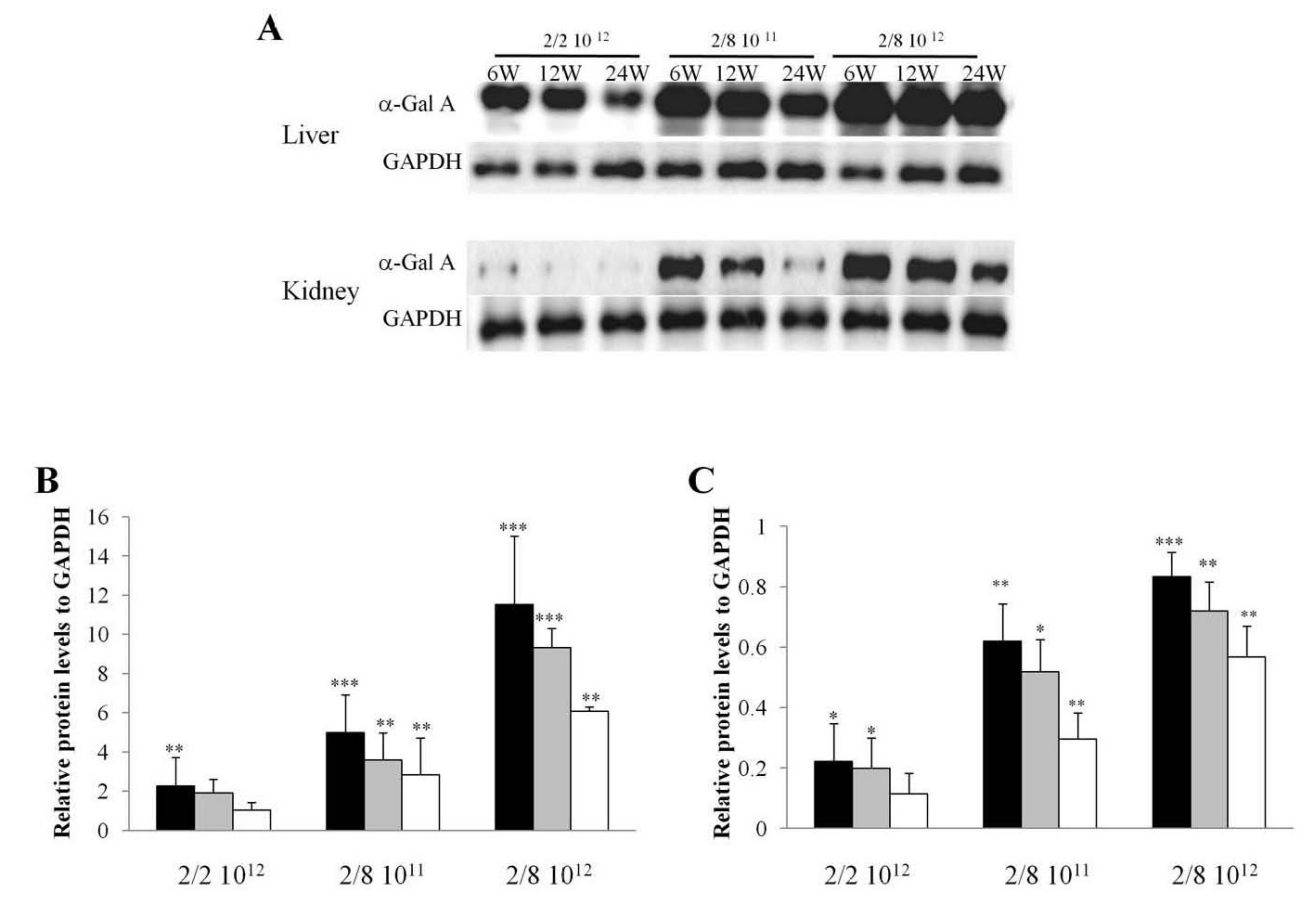
**Western blot analysis of α-galactosidase A expression in liver and kidney at 6, 12, and 24 weeks after treatment in Fabry mice**. Liver and kidney tissue lysates were immunoblotted using anti-α-galactosidase A antibodies. α-Gal A protein expression in liver and kidney at 6, 12, and 24 weeks after injection is demonstrated in (A), and levels of α-Gal A in liver (B) and kidney (C) were quantified using a bioimaging analyzer. Experiments were repeated approximately three to five times using each sample. Values are expressed as means ± SD (*n *= 3 or 4). *p < 0.05, **p < 0.01 and ***p < 0.001 vs. GAPDH using Student's *t *test. Black bar: 6 weeks after injection (*n *= 5), gray bar: 12 weeks after injection (*n *= 3), white bar: 24 weeks after injection (*n *= 3).

The liver α-Gal A content was significantly higher in the mice treated with 2 × 10^12 ^particles of rAAV 2/8-hAGA vector than in the mice treated with 2 × 10^12 ^particles of rAAV 2/2-hAGA vector or 2 × 10^11 ^particles of rAAV 2/8-hAGA vector. The α-Gal A protein levels in the liver showed no significant changes at the various time points (Fig. 2A). The kidney α-Gal A expression levels in the mice treated with 2 × 10^12 ^particles of rAAV 2/8-hAGA vector were the highest (Fig. 2B). However, the expression level was not much different than that in the mice treated with 2 × 10^11 ^particles of rAAV 2/8-hAGA vector. The expression of α-Gal A in the kidney of mice treated with 2 × 10^12 ^particles of rAAV 2/2-hAGA vector was almost undetectable. These results suggest that differences in the viral expression serotype yield different dose titers.

### Liver function test

Liver toxicity was evaluated at 1 week and 6 weeks after tail-vein administration of the rAAV 2/8 vector by measuring ALP, SGPT, and SGOT levels. At 1 week after treatment, mean ALP levels in the untreated Fabry mice and Fabry mice treated with 2 × 10^12 ^particles of rAAV vector were 28 U/L and 30 U/L, respectively. Mean SGOP levels in the untreated Fabry mice were 92 U/L and 102 U/L in the treated Fabry mice. The serum ALP, SGOP, and SGPT levels were not significantly changed at 6 weeks after the treatment.

### Immunohistochemistry of α-Gal A in the kidney

**Figure 3:**
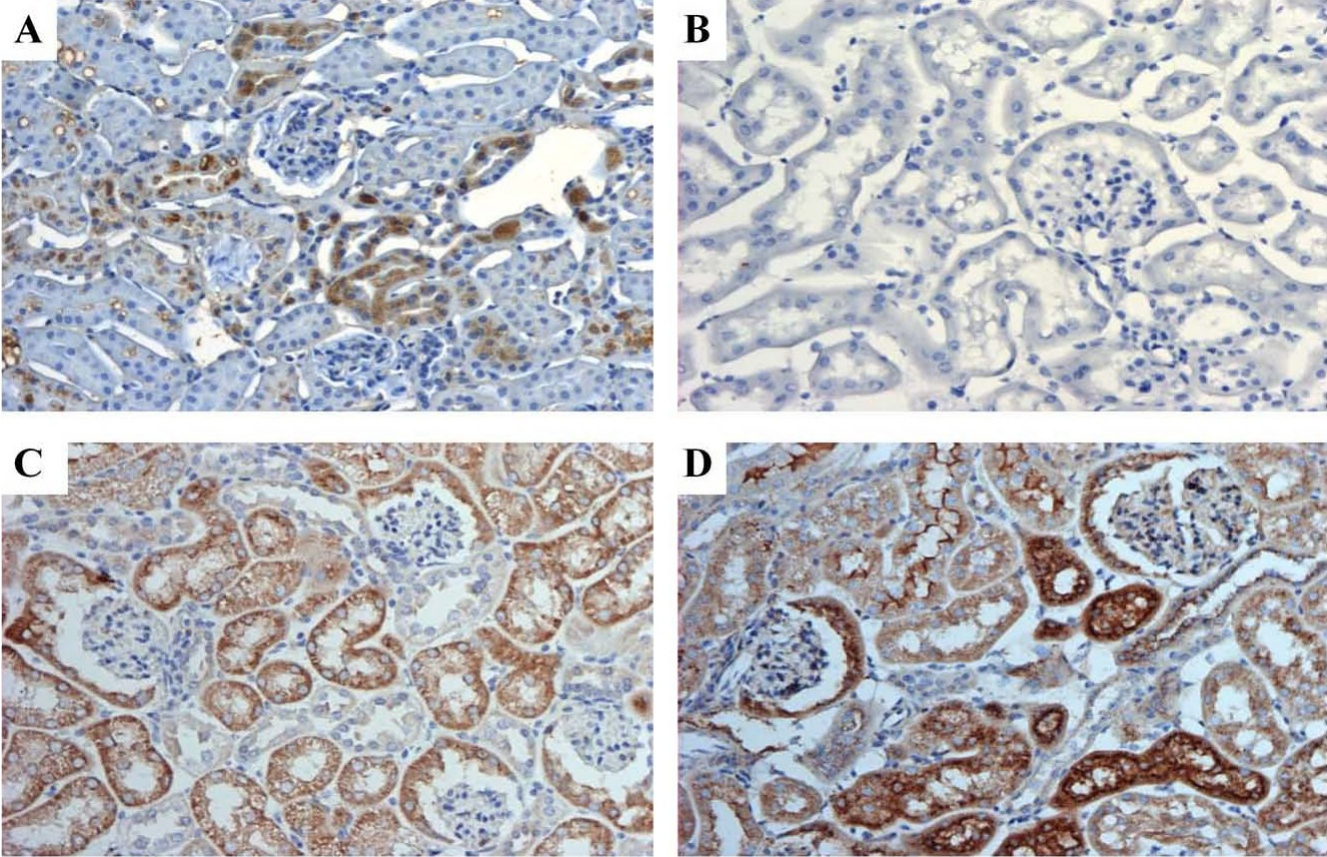
**α-Gal A immunostaining in the kidney**. Kidney sections were stained with peroxidase-conjugated rabbit anti-human α-galactosidase A shown as browning to plasmic staining. (A) Wild-type mice, (B) untreated Fabry mice, (C) Fabry mice treated with ERT, (D) Fabry mice treated with gene therapy (2 × 10^12 ^particles of rAAV2/8-hAGA) (×200).

The kidneys of wild-type mice were strongly labeled with α-Gal A, and most staining was observed in tubular epithelial cells (Fig. 3A). Glomerular cells including podocytes and mesangial cells did not express α-Gal A at detectable levels. However, α-Gal A immunoreactivity was not evident in the Fabry mice (Fig. 3B). The tubules of the glomerulus showed a strong staining pattern and virtually every cell in the vessel wall labeled positive for α-Gal A in the mice that had ERT and mice that had gene therapy (Fig. 3C and 3D).

### Gb3 staining in the kidneys of Fabry mice

**Figure 4:**
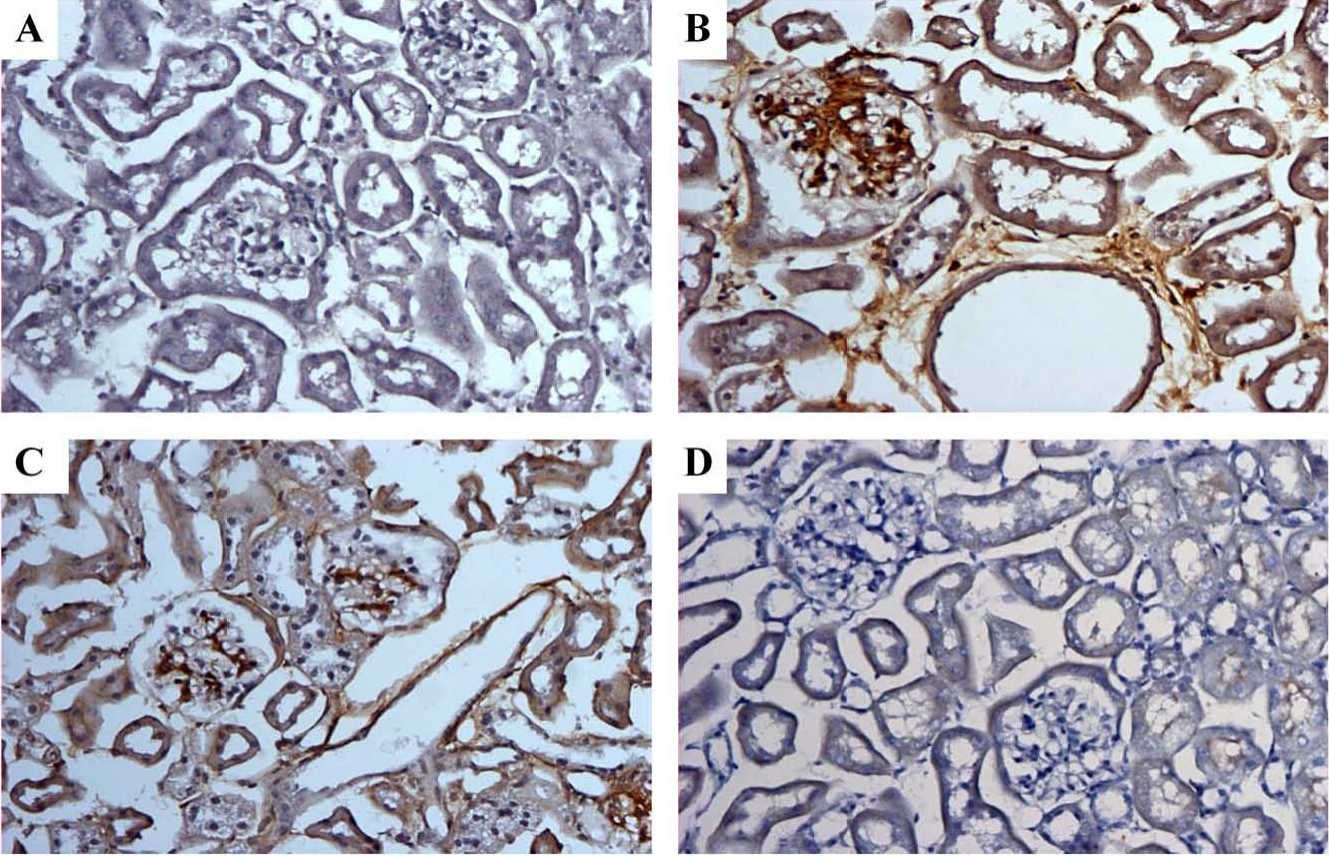
**Immunohistochemistry of CD77/Gb3 in the kidney of Fabry mice**. (A) Wild-type mice unstained, (B) staining appeared in glomeruli and tubules of untreated Fabry mice, (C) stained tubules and glomeruli in Fabry mice treated with ERT, (D) No detection after 6 weeks in Fabry mice injected with 2 × 10^12 ^particles of rAAV2/8-hAGA (×200).

Gb3 immunoreactivity was not observed in wild-type mice (Fig. 4A). However Gb3 immunoreactivity strongly appeared in the kidneys of Fabry mice (Fig. 4B). As expected, Gb3 staining in the kidneys of Fabry mice treated with enzyme replacement showed a mild amelioration of Gb3 deposition in the glomerulus and tubules (Fig. 4C), whereas no Gb3 was detected in the kidneys of mice treated with gene therapy (Fig. 4D). The Gb3 immunostaining signal in the Fabry mice significantly decreased after treatment with either ERT or gene therapy.

### Ultrastructural study of the kidneys of Fabry mice

**Figure 5:**
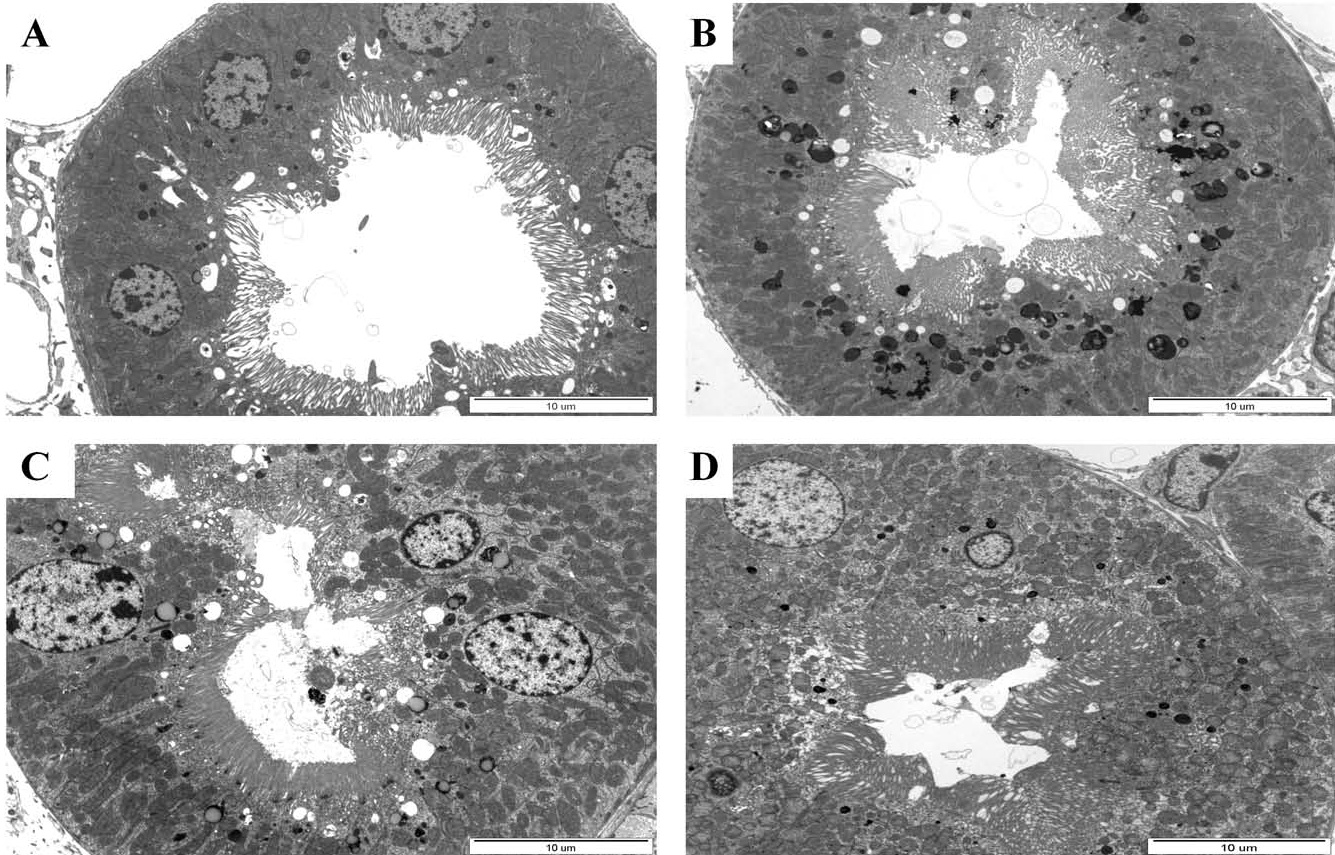
**Gb3 clearance from proximal renal tubules of kidney of rAAV 2/8-treated Fabry mice**. (A) Wild-type mice, (B) Fabry mice (at 24 weeks), (C) 6 weeks after ERT, and (D) 6 weeks after gene therapy (2 × 10^12 ^rAAV2/8-hAGA). The mice were killed 6 weeks after injection and kidney tissue was examined by electron microscopy. Gb3 containing myeloid bodies were recognized in proximal tubules (×8000).

The ultrastructure of the mouse renal proximal tubules was observed by electron microscopy. Gene therapy more effectively removed lipid accumulation from proximal tubules than ERT, shown as a round, dark, laminated intracytoplasmic body (Fig. 5A-D).

**Figure 6:**
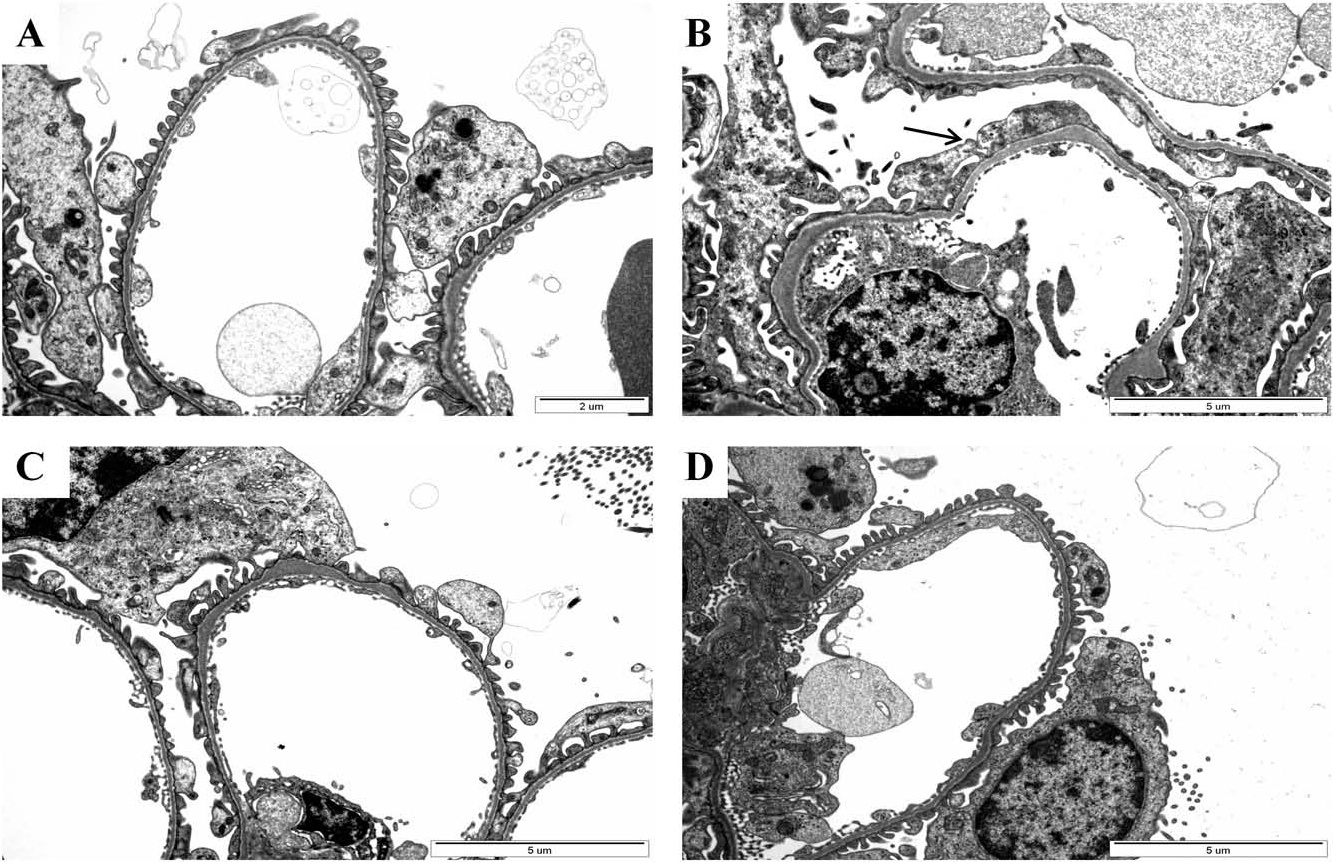
**Ultrastructure of podocytes in the kidney of Fabry mouse**. Compared with wild-type mice (A, × 15000), foot process effacement and thickening of the basement membrane were noted in Fabry mice kidneys (B, × 12000). After 6 weeks ERT (C, × 12000), foot process fusion appeared in a few glomerular podocytes. The glomerular podocytes of kidneys from mice with gene therapy (2 × 10^12 ^particles of rAAV2/8-hAGA) appeared normal (D, ×12000).

The podocytes of wild-type mice, Fabry mice, mice treated with ERT, and mice treated with rAAV-hAGA gene transfer are shown in Fig. 6. In the podocytes of the Fabry mice, foot process fusion and a storage process occurred and Gb3 accumulated, while filtration slits formed multivesicular bodies and degraded, and the slits diaphragm formed a complex (Fig. 6B). When such phenomena occur, proteinuria and glomerulosclerosis can develop. In addition, in the inner capillaries, the pores of endothelial cells underwent fenestration and formed an inclusion. The mesangial cells became complex and began to resemble an inflammatory state. In the Fabry mice treated with ERT, foot process fusions appeared in a few glomerular podocytes (Fig. 6C), suggesting that podocyte injury recovered partially by ERT. The glomerular podocyte of mice kidney treated with gene therapy appeared completely normal (Fig. 6D).

## Discussion

Conventional ERT using recombinant α-Gal A is an effective treatment for Fabry disease. In ERT for Fabry disease, α-Gal A injected intravenously decreases Gb3 accumulation [5-9].

In this study, we sought to determine whether the use of a pseudotyped rAAV 2/8 vector, which purportedly produces more efficient hepatic transduction [14,21], would produce higher levels of α-Gal A expression and consequently greater affects on the pathology in the affected kidneys of Fabry mice. These studies proved that higher levels of enzyme production could be achieved with a recombinant AAV2/8 vector than with an AAV2/2 vector, and that this led to significantly greater and more rapid reduction of lysosomal storage of Gb3 in the kidneys of treated Fabry mice. Thus, whereas the kidneys appear to be somewhat refractory to treatment, this limitation is overcome, at least in part, by exposure to higher levels of the enzyme [22,23]. Gene therapy with a pseudotyped rAAV2/8 vector has the unique potential to provide a safe and long-lasting treatment to overcome the current requirement for chronic frequent enzyme infusions and to treat diseases of the renal endothelial cell. In this study, long-term expression of α-Gal A was observed in the mouse model of Fabry disease for up to 60 weeks after treatment. These findings may be the result of a successful transgenic effect in the establishment of rAAV2/8-hAGA. The rAAV2/8-hAGA transfer via mice tail veins did not result in liver toxicity. A progressive decline in α-Gal A activity was observed in the Fabry mice during the period examined [12]. However, the residual enzyme activity at 60 weeks after treatment in the Fabry mice treated with rAAV2/8-hAGA appeared to be sufficient to maintain to correct the Gb3 levels in the tissues. A progressive decline in the transgene expression may reflect the characteristics of the rAAV vectors, which exist primarily as extrachromosomal elements [24,25], or development of an immune response to human α-Gal A protein in α-Gal A null mice [12,26].

Previous studies indicated the overexpression of human α-galactosidase A, as well as the existence of the α-Gal A gene in the responsible organs [27-29]. The α-Gal A expression is observed in all tubular segments and interstitial cells of normal kidneys [29]. A previous study indicated that although the glycosphingolipids may accumulate in endothelial, glomerular, and tubular cells in Fabry disease, glomeruli and endothelial cells did not express the enzyme after ERT [29]. The immunohistochemical analysis in this present study clarified that α-Gal A expression is observed in glomeruli of the kidneys of Fabry mice after high-dose gene therapy. In accordance with a previous study [29], no α-Gal A was detected in the glomeruli after ERT. The α-Gal A protein expressed in glomeruli might arise from protein secreted by the liver, a depot organ in Fabry mice for the delivery of recombinant enzyme, rather than direct transduction of rAAV 2/8 [12,14,30].

The ultrastructure of mice kidneys was examined by electron microscopy. Proximal tubules (Fig. 6) in mice treated by gene therapy more effectively removed lipid accumulation than those in mice treated by ERT. Glomerular changes, including segmental sclerosis, focal foot process fusions, and endothelial microlesions, were detected by transmission electron microscopy. Proteinuria has been described as the first sign of renal functional impairment in Fabry disease [31-33]. Although not completely understood, it seems likely that patients with Fabry disease have a predisposition to inflammatory or immune-mediated renal disease related to the toxic accumulation of glycosphingolipids and exposure of the glomerular basement with consequent synechiae formation [22,23,34]. This accumulation is seen in patients at renal biopsy, with no other findings that suggested alternative causes of nephrotic syndrome, although the foot process fusion seen on electron microscopy can also be seen with minimal change in disease. Clinical studies of ERT for Fabry disease have demonstrated different degrees of clearance of glycosphingolipid deposits. This clearance results in improved glomerular architecture over several months of therapy, but has a limited effect on proteinuria [15,35]. The rate of reaccumulation of Gb3 after injection of 2 × 10^12 ^viral particles per mouse was assessed to determine the dose frequency needed to maintain reduced Gb3 levels. The accumulated hepatic Gb3 was rapidly cleared and remained at undetectable levels for 6 weeks, whereas the spleen and cardiac Gb3 concentrations were maximally decreased at 6 and 12 weeks postinjection, respectively, before both began to reaccumulate. This finding suggests that a dose of 2 × 10^12 ^viral particles per mouse could both deplete the accumulated Gb3 and prevent its reaccumulation. The biochemical demonstration of depletion of accumulated tissue Gb3 is consistent with the ultrastructural findings of fewer, smaller, or less dense lysosomes in the tissues of treated mice. Markedly decreased lysosomal glycolipid storage was observed in podocytes and tubules of the kidneys. These findings suggest that α-Gal A is readily endocytosed into endosomes for subsequent processing by lysosomes containing the substrate.

There are several issues to overcome before rAAV vector-mediated gene therapy can be used clinically. Efficient and versatile large-scale AAV vector-production systems are needed for clinical application of this vector [36]. The host immune response remains of concern [25]. Although AAV vectors are unlikely to cause insertional mutagenesis, the issue remains of concern; however, the recombinant AAV genome does not integrate site-specifically into the chromosome [24,25]. Despite these concerns, AAV remains a promising delivery system for gene therapy. For the mouse model of Fabry disease, a high dose of rAAV-mediated α-Gal A gene transfer achieved a greater efficacy than did ERT. A single injection of rAAV2/8-hAGA in Fabry mice produced long-term efficacy and caused no apparent hepatic damage. Immunohistochemistry and electron microscopy studies showed clear evidence of effective α-Gal A expression and Gb3 clearance in the kidney of Fabry mice given gene therapy. Although it is difficult to conclude which system is more effective for treating patients with Fabry disease, AAV-mediated gene therapy can be an effective therapeutic strategy.

## Conclusions

These studies have shown the efficacy of rAAV 2/8-hAGA-mediated gene therapy for both biochemical and functional deficits in the Fabry disease mouse model. Recombinant AAV 2/8-hAGA-mediated expression produced good efficacy that was comparable to that of ERT, especially in the kidney.

## Competing interests

The authors declare that they have no competing interests.

## Authors' contributions

JOC: performed most experiments including mouse care. MHL: prepared viral vectors and mouse care. SCJ: designed the experiments and interpreted the results. JOC, HYP, and SCJ: general discussion and work on manuscript.

## References

[B1] Brady RO Gal AE Bradley RM Martensson E Warshaw AL Laster L Enzymatic defect in Fabry's disease: ceramidetrihexosidase deficiency N Engl J Med 1967 276 1163 1167 6023233 10.1056/NEJM196705252762101

[B2] Brady RO Enzyme replacement for lysosomal diseases Annu Rev Med 2006 57 283 296 10.1146/annurev.med.57.110104.115650 16409150

[B3] Desnick RJ Brady RO Fabry disease in childhood J Pediatr 2004 144 S20 26 10.1016/j.jpeds.2004.01.051 15126980

[B4] DeVeber GA Schwarting GA Kolodny EH Kowall NW Fabry disease: immune-cytochemical characterization of neuronal involvement Ann Neurol 1992 31 409 415 10.1002/ana.410310410 1375013

[B5] Schiffmann R Murray GJ Treco D Daniel P Sellos-Moura M Myers M Quirk JM Zirzow GC Borowski M Loveday K Infusion of α-galactosidase A reduces tissue globotriaosylceramide storage in patients with Fabry disease Proc Natl Acad Sci USA 2000 97 365 370 10.1073/pnas.97.1.365 10618424 PMC26669

[B6] Eng CM Guffon N Wilcox WR Germain DP Lee P Waldek S Caplan L Linthorst GE Desnick RJ Safety and efficacy of recombinant human alpha-galactosidase A replacement therapy in Fabry's disease N Engl J Med 2001 345 9 16 10.1056/NEJM200107053450102 11439963

[B7] Lee K Jin X Zhang K Copertino L Andrews L Baker-Malcolm J Geagan L Qiu H Seiger K Barngrover D McPherson JM A biochemical and pharmacological comparison of enzyme replacement therapies for the glycolipid storage disorder Fabry disease Glycobiology 2003 13 305 313 10.1093/glycob/cwg034 12626384

[B8] Schiffmann R Kopp JB Austin HA Sabnis S Moore DF Weibel T Balow JE Brady RO Enzyme replacement therapy in Fabry disease: a randomized controlled trial JAMA 2001 285 2743 2749 10.1001/jama.285.21.2743 11386930

[B9] Germain DP Waldek S Banikazemi M Bushinsky DA Charrow J Desnick RJ Lee P Loew T Vedder AC Abichandani R Wilcox WR Guffon N Sustained, long-term renal stabilization after 54 months of agalsidase beta therapy in patients with Fabry disease J Am Soc Nephrol 2007 18 1547 1557 10.1681/ASN.2006080816 17409312

[B10] Takenaka T Qin G Brady RO Medin JA Circulating alpha-galactosidase A derived from transduced bone marrow cells: relevance for corrective gene transfer for Fabry disease Hum Gene Ther 1999 10 1931 1939 10.1089/10430349950017293 10466627

[B11] Ziegler RJ Yew NS Li C Cherry M Berthelette P Romanczuk H Ioannou YA Zeidner KM Desnick RJ Cheng SH Correction of enzymatic and lysosomal storage defects in Fabry mice by adenovirus-mediated gene transfer Hum Gene Ther 1999 10 1667 1682 10.1089/10430349950017671 10428212

[B12] Jung SC Han IP Limaye A Xu R Gelderman MP Zerfas P Tirumalai K Murray GJ During MJ Brady RO Qasba P Adeno-associated viral vector-mediated gene transfer results in long-term enzymatic and functional correction in multiple organs of Fabry mice Proc Natl Acad Sci USA 2001 98 2676 2681 10.1073/pnas.051634498 11226298 PMC30197

[B13] Ziegler RJ Cherry M Barbon CM Li C Bercury SD Armentano D Desnick RJ Cheng SH Correction of the biochemical and functional deficits in Fabry mice following AAV8-mediated hepatic expression of alpha-galactosidase A Mol Ther 2007 15 492 500 10.1038/sj.mt.6300066 17191071

[B14] Gao G Lu Y Calcedo R Grant RL Bell P Wang L Figueredo J Lock M Wilson JM Biology of AAV serotype vectors in liver-directed gene transfer to nonhuman primates Mol Ther 2006 13 77 87 10.1016/j.ymthe.2005.08.017 16219492

[B15] Branton MH Schiffmann R Sabnis SG Murray GJ Quirk JM Altarescu G Goldfarb L Brady RO Balow JE Austin Iii HA Kopp JB Natural history of Fabry renal disease: influence of alpha-galactosidase A activity and genetic mutations on clinical course Medicine 2002 81 122 138 10.1097/00005792-200203000-00003 11889412

[B16] Ohshima T Murray GJ Swaim WD Longenecker G Quirk JM Cardarelli CO Sugimoto Y Pastan I Gottesman MM Brady RO Kulkarni AB α-Galactosidase A deficient mice: A model of Fabry disease Proc Natl Acad Sci USA 1997 94 2540 2544 10.1073/pnas.94.6.2540 9122231 PMC20124

[B17] Ioannou YA Zeidner KM Gordon RE Desnick RJ Fabry disease: preclinical studies demonstrate the effectiveness of alpha-galactosidase A replacement in enzyme-deficient mice Am J Hum Genet 2001 68 14 25 10.1086/316953 11115376 PMC1234907

[B18] Kusiak JW Quirk JM Brady RO Purification and properties of the two major isozymes of α-galactosidase from human placenta J Biol Chem 1978 253 184 190 201618

[B19] Rose HG Oklander M Improved procedure for the extraction of lipids from human erythrocytes J Lipid Res 1965 6 428 431 14336214

[B20] Shahi SK Ranga S Khurana SK Talib VH Free/total prostate specific antigen ratio: a new hope Indian J Pathol Microbiol 1999 42 1 2 10420677

[B21] Nakai H Fuess S Storm TA Muramatsu S Nara Y Kay MA Unrestricted hepatocyte transduction with adeno-associated virus serotype 8 vectors in mice J Virol 2005 79 214 224 10.1128/JVI.79.1.214-224.2005 15596817 PMC538708

[B22] Pisani A Spinelli L Sabbatini M Andreucci MV Procaccini D Abbaterusso C Pasquali S Savoldi S Comotti C Cianciaruso B Enzyme replacement therapy in Fabry disease patients undergoing dialysis: effects on quality of life and organ involvement Am J Kidney Dis 2005 46 120 127 10.1053/j.ajkd.2005.03.016 15983965

[B23] Alroy J Sabnis S Kopp JB Renal pathology in Fabry disease J Am Soc Nephrol 2002 Suppl 2 S134 138 12068025

[B24] Nakai H Storm TA Kay MA Recruitment of single-stranded recombinant adeno-associated virus vector genomes and intermolecular recombination are responsible for stable transduction of liver in vivo J Virol 2000 74 9451 9463 10.1128/JVI.74.20.9451-9463.2000 11000214 PMC112374

[B25] Daya S Berns KI Gene therapy using adeno-associated virus vectors Clin Microbiol Rev 2008 21 583 593 10.1128/CMR.00008-08 18854481 PMC2570152

[B26] Ziegler RJ Lonning SM Armentano D Li C Souza DW Cherry M Ford C Barbon CM Desnick RJ Gao G Wilson JM Peluso R Godwin S Carter BJ Gregory RJ Wadsworth SC Cheng SH AAV2 vector harboring a liver-restricted promoter facilitates sustained expression of therapeutic levels of alpha-galactosidase A and the induction of immune tolerance in Fabry mice Mol Ther 2004 9 231 240 10.1016/j.ymthe.2003.11.015 14759807

[B27] Shimmoto M Kase R Itoh K Utsumi K Ishii S Taya C Yonekawa H Sakuraba H Generation and characterization of transgenic mice expressing a human mutant alpha-galactosidase with an R301Q substitution causing a variant form of Fabry disease Vision Res 1997 48 353 359 10.1016/s0014-5793(97)01263-5 9395081

[B28] Kase R Shimmoto M Itoh K Utsumi K Kotani M Taya C Yonekawa H Sakuraba H Immunohistochemical characterization of transgenic mice highly expressing human lysosomal alpha-galactosidase Biochim Biophys Acta 1998 1406 260 266 9630664 10.1016/s0925-4439(98)00012-x

[B29] Christensen EI Zhou Q Sørensen SS Rasmussen AK Jacobsen C Feldt-Rasmussen U Nielsen R Distribution of alpha-galactosidase A in normal human kidney and renal accumulation and distribution of recombinant alpha-galactosidase A in Fabry mice J Am Soc Nephrol 2007 18 698 706 10.1681/ASN.2006080822 17287429

[B30] Neufeld EF Lysosomal storage diseases Annu Rev Biochem 1991 60 257 280 10.1146/annurev.bi.60.070191.001353 1883197

[B31] Branton M Schiffmann R Kopp JB Natural history and treatment of renal involvement in Fabry disease J Am Soc Nephrol 2002 13 S139 143 12068026

[B32] Ortiz A Oliveira JP Wanner C Recommendations and guidelines for the diagnosis and treatment of Fabry nephropathy in adults Nat Clin Pract Nephrol 2008 4 327 336 10.1038/ncpneph0806 18431378

[B33] Fervenza FC Torra R Warnock DG Safety and efficacy of enzyme replacement therapy in the nephropathy of Fabry disease Biologics 2008 2 823 843 19707461 10.2147/btt.s3770 PMC2727881

[B34] Kawamura O Sakuraba H Itoh K Suzuki Y Doi M Kuwabara H Oshima S Abe S Warabi H Yoshizawa N Subclinical Fabry's disease occurring in the context of IgA nephropathy Clin Nephrol 1997 47 71 75 9049452

[B35] Tahir H Jackson LL Warnock DG Antiproteinuric therapy and fabry nephropathy: sustained reduction of proteinuria in patients receiving enzyme replacement therapy with agalsidase-beta J Am Soc Nephrol 2007 18 2609 2617 10.1681/ASN.2006121400 17656478

[B36] Clément N Knop DR Byrne BJ Large-scale adeno-associated viral vector production using a herpesvirus-based system enables manufacturing for clinical studies Hum Gene Ther 2009 20 796 806 10.1089/hum.2009.094 19569968 PMC2861951

